# Tuberculosis contact investigation: an evaluation of yield and guideline adherence, Upper Bavaria, Germany, 2018 to 2022

**DOI:** 10.2807/1560-7917.ES.2025.30.39.2500096

**Published:** 2025-10-02

**Authors:** Sarah van de Berg, Andreas Beyerlein, Johannes Stephani, Beatrix Bäumler-Merl, Manuela Jäger, Dagmar Königer, Ruprecht Schmidt-Ott

**Affiliations:** 1 Bavarian Health and Food Safety Authority, Oberschleißheim, Germany; 2 Landratsamt Rosenheim, Staatliches Gesundheitsamt, Rosenheim, Germany; 3 Landratsamt Starnberg, Gesundheitsamt, Starnberg, Germany; 4 Landratsamt Dachau, Gesundheitsamt, Dachau, Germany; 5 Regierung von Oberbayern, Munich, Germany

**Keywords:** tuberculosis, contact investigation, contact tracing, latent tuberculosis infection, *Mycobacterium tuberculosis* infection, testing coverage, yield

## Abstract

**BACKGROUND:**

Contact investigation of index cases is important for tuberculosis (TB) control in low-incidence countries, yet key performance metrics have not been evaluated in Germany.

**AIM:**

We aimed to assess the yield of TB contact investigations, compliance with national guidelines and risk factors for infection among contacts.

**METHODS:**

We analysed anonymised data of TB patients and their contacts collected between 2018 and 2022 in Upper Bavaria, Germany. We assessed testing coverage, latent TB infection (LTBI), TB yield, primary prophylaxis and preventive treatment coverage. Risk factors for *M. tuberculosis* infection (positive tuberculin skin test (TST) / interferon-gamma release assay (IGRA) and/or TB diagnosis) among contacts were identified using multivariable logistic regression and classification tree.

**RESULTS:**

Of the 2,186 contacts of 174 TB patients, 2,022 (92.5%) had a valid TST/IGRA result and/or a TB diagnosis. Of these, 308 (15.2%) had *M. tuberculosis* infection, including 10 (0.5%) with TB. Of 241 contacts with LTBI, 66 (28.2%) completed preventive treatment. Among 124 children < 5 years, testing coverage was 75.8%, 16.9% received an immediate chest X-ray and 72.7% primary prophylaxis. Key predictors of infection were born outside Germany (odds ratio (OR) = 2.85; 95% confidence interval (CI): 1.94–4.21) and exposure in community housing (OR = 2.65; 95% CI: 1.65–4.30; reference: exposure at work) or household/family (OR = 2.62; 95% CI: 1.74–4.00).

**CONCLUSION:**

We observed high screening coverage of contacts and yield and risk factors comparable to other low-incidence settings. There is room for improvement regarding preventive treatment and screening of children < 5 years.

**Key public health message**

**What did you want to address in this study and why?**

Timely investigation of contact persons from patients with tuberculosis (TB) is key for limiting the spread of the disease. We wanted to explore compliance with current national guidelines and to assess the efficiency of current practice of contact tracing in detecting *Mycobacterium tuberculosis* infection and TB disease. We also aimed to assess risk factors for infection among contacts to guide public health policies.

**What have we learnt from this study?**

We found that testing coverage among contacts was high, indicating overall good compliance with German guidelines. However, we saw gaps in the treatment of infected contact persons to prevent disease progression. We also found that screening needs to be improved for children under 5 years. Risk factors for infection were comparable to other low TB incidence settings.

**What are the implications of your findings for public health?**

Our findings highlight gaps in the current practice of contact investigation that need to be addressed, particularly for children under 5 years of age. Our results may also guide resource allocation when contact tracing capacities are limited, e.g. under pandemic conditions. Overall, monitoring and evaluating TB contact investigations is essential to identify areas for improvement and ensure practices are adapted to maximise their effectiveness.

## Introduction

Humans infected with *Mycobacterium tuberculosis* may develop acute tuberculosis (TB) or latent TB infection (LTBI) without signs of acute disease, with an estimated lifetime progression risk of 5–10% [1,2]. In Germany, TB is a rare disease with an annual incidence ranging from 4.7 to 7.2 cases per 100,000 inhabitants between 2012 and 2022 [3].

As in other countries with a low TB incidence, defined as fewer than 10 cases per 100,000 population per year [4], contact investigation of index cases is a key component of TB control [5]. It aims to (i) reduce TB morbidity and fatality and prevent further transmission by timely identifying and treating source and secondary cases, and (ii) prevent future TB cases by detecting and treating LTBI among contacts [6]. Efficient contact investigation relies on a risk-based approach, focusing resources on contacts who are at substantial infection risk, while ensuring no secondary LTBI or TB is overlooked.

Commonly, TB contact investigation is evaluated based on the yield of LTBI and TB among contacts. Further indicators include the proportion of contacts tested for LTBI and TB and the proportion of infected contacts completing preventive treatment [6]. In a recent systematic review of contact tracing studies in low-incidence countries [7], the yield varied largely by setting, possibly due to differences in TB epidemiology and screening procedures. To date, only a few studies have assessed contact tracing in Germany [8-11].

This study evaluates TB contact investigation data collected in Upper Bavaria, Germany, over 5 years to enhance our understanding of TB transmission and to improve the efficiency of TB contact investigation in this low-incidence setting. We assessed *M. tuberculosis* infection and TB yield among close contacts, explored risk factors for infection and evaluated compliance with national guidelines for contact identification, screening and primary prophylaxis/preventive treatment.

## Methods

### Study population and data collection

We acquired anonymised TB contact investigation data collected between 2018 and 2022 by 10 of 22 local public health departments in Upper Bavaria, Germany. The participating 10 districts had a total population of about ca 1.5 million inhabitants, thus 11.3% of the whole population of Bavaria as of 31 December 2022 [12]. In Germany, TB is a notifiable disease; clinicians and laboratories report cases to local public health departments who, in Bavaria, forward the information to the Bavarian Health and Food Safety Authority (Bayerisches Landesamt für Gesundheit und Lebensmittelsicherheit, LGL).

Index cases were included if they had at least one close contact traced by the same local public health department. Local public health departments collected detailed information only on contacts living within their respective district. Contacts living outside these districts were therefore not included in our dataset. Eight of the 10 local public health departments provided the number of contacts residing in other districts, along with their exposure settings.

Contact investigation of patients with TB in Germany follows national guidelines [13]. Infectious TB patients are asked to provide information on close contacts. Eligible contacts are invited by local public health departments for clinical examination and diagnostic testing. Contact selection considers the infectiousness of the index case along with cumulative contact time and intensity. Detailed criteria for contact selection are provided in Supplement S1. Close contacts are offered tuberculin skin test (TST) or interferon-gamma release assay (IGRA) 8 weeks after their last exposure to the index patient. Contacts younger than 15 years are additionally offered immediate clinical examination and TST/IGRA, while contacts younger than 5 years, or immuno-compromised contacts, are also recommended immediate chest X-ray examination and primary prophylaxis. Positive TST/IGRA results prompt diagnostics for TB including clinical, sputum and chest X-ray examination. Contacts with LTBI, i.e. positive TST/IGRA result without concomitant signs of acute TB, are offered preventive treatment according to relevant guidelines [14,15]. Preventive treatment and follow-up is generally provided by family physicians, pulmonologists or infectious diseases specialists. Follow-up of contacts with LTBI includes at least one control chest X-ray after completed preventive treatment or, if no treatment was initiated, within a year. Treatment (regimen, uptake and completion) as well as chest X-ray outcomes are to be reported to the local public health office. In Germany, recommended regimens for preventive therapy are rifampicin for 4 months (4R), rifampicin plus isoniazid for 3 months (3HR) or isoniazid for 9 months (9H) [14].

For TB index cases, we collected information on sex (male/female), age (in years), country of birth (in or outside Germany), microbiological test results of respiratory specimens (sputum, bronchoalveolar lavage, bronchial secretions, or gastric juice), smear microscopy (positive/negative), culture (positive/negative), polymerase chain reaction (PCR) (positive/negative) and findings from medical imaging (TB-typical changes with cavity/cavities, TB-typical changes without cavities, non-specific changes, normal). Based on this information, infectiousness of index cases was categorised as either low (only culture or PCR-positive), moderate (smear-positive without visible cavities on chest X-ray examination) or high (smear-positive with visible cavity/cavities). Microbiological results also included information on drug resistance of the *M. tuberculosis* isolate (sensitive, multidrug-resistant (MDR) or pre-extensively drug-resistant (pre-XDR)).

Data collected from close contacts of TB index cases included sex (male/female), age (< 5, 5–14, 15–49 or ≥ 50 years), country of birth (in or outside Germany), number of years of residence in Germany (for those born outside Germany; < 2, 2–9, 10–29 or ≥ 30 years), assumed duration of exposure (< 8, 8–40 or > 40 h), TST/IGRA results, where applicable, information on initiation and completion of preventive treatment, follow-up (clinical and chest X-ray examination results) and diagnosis of TB. Further, information about the exposure setting was collected and recoded into six categories: (i) community housing (household-like settings such as refugee or homeless shelters), (ii) day care/school, (iii) household/family (including family and partners not living in the same household), (iv) patient/nursing care (as patient), (v) social activities, or (vi) work (including work in medical or nursing facilities). Contacts were defined as infected with *M. tuberculosis* if they had a positive TST or IGRA test result or were diagnosed with TB during the follow-up period. Contacts were classified as having LTBI if they had a positive TST or IGRA result and a normal chest X-ray.

### Statistical analysis

Using descriptive analysis, we assessed the demographic characteristics of TB index cases and their close contacts. In addition, we compared the index cases in our dataset with all infectious pulmonary TB cases notified in Bavaria (LGL notification dataset). In the latter, we included open pulmonary TB patients, i.e. patients fulfilling the national reference definition for TB [16] if they were also positive for smear microscopy, culture and/or PCR of respiratory material. We applied chi-square and Fisher's exact tests to investigate differences between cases in the contact tracing dataset and cases in the LGL notification dataset from non-participating districts.

We used the contact tracing dataset to determine the proportion of contacts tested for LTBI (testing coverage), the proportion of *M. tuberculosis* infection, LTBI and TB, respectively, among contacts (yield) as well as the proportion of contacts identified with LTBI who completed preventive treatment (treatment coverage), overall and stratified by contact characteristics.

To investigate potential predictors of *M. tuberculosis* infection in contacts, we included the following variables in a logistic regression analysis: age, sex and origin of the contact person, exposure time and exposure setting, as well as country of birth and infectiousness of the corresponding index case. Observations with missing values about *M. tuberculosis* infection or any of the predictor variables were excluded from this analysis. Further, a classification tree was calculated based on the same risk factors, with the addition of the number of years a contact person born outside Germany had been resident in Germany. In the classification tree analysis, observations with missing values about *M. tuberculosis* infection in contact persons were omitted, while observations with missing values about any of the predictor variables were imputed using surrogate variables [17]. Splits were only chosen if they decreased the overall lack of fit by a factor of 0.005. The minimum number of observations per node was set to the square root of the initial sample size [18,19].

The statistical analyses were carried out in R software version 4.4.1 (R Foundation, Vienna, Austria) using the *rpart* package. Statistical significance was determined by a level of 0.05 without adjustment for multiple testing.

## Results

### Description of index patients

Contact investigation data on 174 TB index patients were collected, 132 of whom were born outside Germany (Table 1). Most German-born patients (33/40) were aged 50 years or older, while two thirds of non-German-born (86/132) were aged 15–49 years. More than half of the index patients with a known sputum smear result (100/172) were smear-positive, of which 61 could be classified as moderately infectious and 35 as highly infectious, while for four index patients no chest X-ray examination results were available. Eleven had MDR or pre-XDR TB. Three index cases were diagnosed at the end of 2017, but their contact tracing was performed in 2018.

**Table 1:** Description of index patients with active pulmonary tuberculosis, selected districts of Upper Bavaria^a^, 2018–2022 (n = 174)

Patient characteristics	Overall		Born in Germany		Born outside Germany	
	n	%	n	%	n	%
Total	174	100	40	100	132	100
Country of birth						
Germany	40	23.3	NA		NA	
Outside Germany	132	76.7				
Unknown	2	NC				
Sex						
Male	118	67.8	32	80.0	84	63.6
Female	56	32.2	8	20.0	48	36.4
Age category (years)						
0–4	1	0.6	0	0	1	0.8
5–14	1	0.6	1	2.5	0	0
15–49	92	53.2	6	15.0	86	65.2
≥ 50	79	45.7	33	82.5	45	34.1
Unknown	1	NC	0	NC	1	NC
Year of notification						
2017	3	1.7	1	2.5	2	1.5
2018	29	16.7	4	10.0	25	18.9
2019	44	25.3	11	27.5	32	24.2
2020	40	23.0	7	17.5	33	25.0
2021	30	17.2	8	20.0	21	15.9
2022	28	16.1	9	22.5	19	14.4
Infectiousness^b^						
Low	72	42.9	19	47.5	53	42.1
Moderate	61	36.3	14	35.0	46	36.5
High	35	20.8	7	17.5	27	21.4
Unknown	6	NC	0	NC	6	NC
Drug resistance						
Sensitive	153	93.3	38	95.0	113	92.6
MDR	7	4.3	1	2.5	6	4.9
pre-XDR	4	2.4	1	2.5	3	2.5
Unknown	10	NC	0	NC	10	NC

MDR: multidrug-resistant; NA: not applicable; NC: not calculated; pre-XDR: pre-extensively drug-resistant.

^a^ Bad Tölz-Wolfratshausen, Berchtesgadener Land, Dachau, City of Ingolstadt, Landsberg am Lech, Mühldorf, Pfaffenhofen, Rosenheim, Starnberg and Weilheim-Schongau.

^b^ Infectiousness was categorised as either low (only culture or PCR positive) or moderate/high (smear-positive without/with visible caverna/ae on medical imaging).

Percentages denote column percent of non-missing values.

The 171 index cases diagnosed with TB between 2018 and 2022 represented 8.2% of all 2,097 TB cases with open pulmonary TB reported in Bavaria within this period and 84.7% of the 202 reported cases in the participating districts. Compared with cases in non-participating districts, there was a significantly higher proportion of study index cases born abroad (76.9% vs 69.6%; chi-square test: p = 0.047) and with MDR or pre-XDR-TB (4.3% vs 2.9%; 2.5% vs 0.4%; Fisher’s exact test: p = 0.007). Supplementary Table S1 provides a comparison between index patients included in the study and pulmonary TB cases notified to the LGL between 2018 and 2022.

### Description of contacts

**Figure 1:**
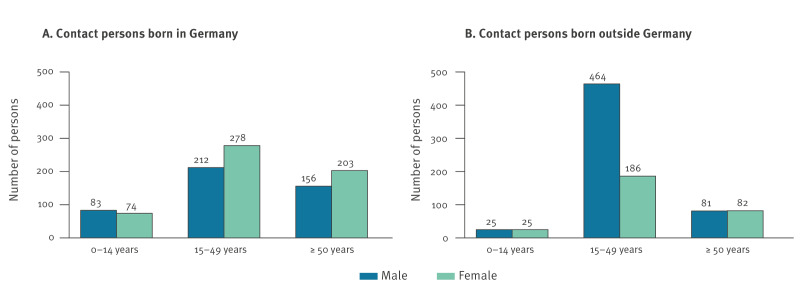
Sex and age distribution of contact persons of index patients with active pulmonary tuberculosis, stratified by country of origin (if available), selected districts of Upper Bavaria^a^, 2018–2022

^a^ Bad Tölz-Wolfratshausen, Berchtesgadener Land, Dachau, City of Ingolstadt, Landsberg am Lech, Mühldorf, Pfaffenhofen, Rosenheim, Starnberg and Weilheim-Schongau.

Data from 2,186 contacts of the 174 TB index patients were available for analysis. Almost half of the contacts with known country of origin (863/1,869) were born outside Germany (Table 2). The distributions of age and sex differed significantly (chi-square test: p < 0.0001 each) between contacts born abroad and contacts born in Germany, with those born abroad being predominantly younger adult males (Figure 1).

**Table 2:** Description of contact persons of index patients with active pulmonary tuberculosis, selected districts in Upper Bavaria^a^, 2018–2022 (n = 2,186)

Characteristics	Overall		Born in Germany		Born outside Germany	
	n	%	n	%	n	%
Total	2,186	100	1006	100	863	100
Country of birth						
Germany	1,006	53.8	NA		NA	
Outside Germany	863	46.2				
Unknown	317	NC				
*Mycobacterium tuberculosis* infection						
Yes	308	15.2	78	8.3	208	26.2
No	1,714	84.8	867	91.7	585	73.8
Unknown	164	NC	61	NC	70	NC
Sex						
Male	1,181	54.1	451	44.8	570	66.0
Female	1,004	45.9	555	55.2	293	34.0
Unknown	1	NC	0	NC	1	NC
Age category (years)						
0–4	124	5.7	85	8.4	14	1.6
5–14	112	5.1	72	7.2	36	4.2
15–49	1,321	60.4	490	48.7	650	75.3
≥ 50	629	28.8	359	35.7	163	18.9
Duration of exposure (hours)						
< 8	290	13.7	199	20.3	87	10.6
8–40	631	29.8	286	29.2	196	23.8
> 40	1,198	56.5	495	50.5	539	65.6
Unknown	67	NC	26	NC	41	NC
Exposure setting						
Community housing	246	11.3	19	1.9	226	26.2
Day care/school	183	8.4	99	9.9	58	6.7
Household/family	582	26.7	259	25.8	284	32.9
Patient/nursing care	177	8.1	92	9.2	17	2.0
Social activities	288	13.2	130	13.0	100	11.6
Work	706	32.4	403	40.2	178	20.6
Unknown	4	NC	4	NC	0	NC

NA: not applicable; NC: not calculated.

^a^ Bad Tölz-Wolfratshausen, Berchtesgadener Land, Dachau, City of Ingolstadt, Landsberg am Lech, Mühldorf, Pfaffenhofen, Rosenheim, Starnberg and Weilheim-Schongau.

Percentages denote column percent of non-missing values.

### Number of investigated contacts per index patient

Overall, there was a median number of 7.5 (interquartile range (IQR): 4–16) investigated contacts per TB index patient in the responsibility of the local public health department. The number of investigated contacts per index person was not significantly different between male (median: 8; IQR: 4–16) and female (median: 7; IQR: 3–14.25) index patients (Mann–Whitney-U test: p = 0.34), or between index patients born in Germany (median: 7; IQR: 3.75–16) or outside Germany (median: 8; IQR: 4–16; p = 0.89), or for index patients with high (median: 10; IQR: 4.5–14.5) compared with moderate (median: 9; IQR: 4–17; p = 0.94) or low infectiousness (median: 6.5; IQR: 2.75–14; p = 0.27). However, the median number of investigated contacts per index person was significantly higher for index patients aged 15–49 years (median: 10; IQR: 5–18.25) than aged 50 years or older (median: 6; IQR: 3–13.5; p = 0.008).

### Number of contacts transferred to other districts

Eight of the 10 local public health departments provided information on the number of contacts transferred to local public health departments from other districts (in total 759 contacts) resulting in a median of four (IQR: 2–8) contacts per index case. These were mainly associated with exposure at work (332 contacts), day care/school (189 contacts), family/partner living separately (96 contacts) and patient/nursing care (70 contacts). In Supplementary Table S2, we append the number of contacts transferred to other districts by exposure setting.

### Testing coverage, yield and treatment coverage among contacts

Among the 2,186 contacts, 2,021 (92.5%) had a valid TST/IGRA result. Taking into account an additional contact who was diagnosed with TB during follow-up, 308 of 2,022 contacts (15.2%) were found to have an *M. tuberculosis* infection. Within 8 weeks after their last exposure to the index patient, 241 contacts (11.9% of 2,022) were diagnosed with LTBI and four with TB. The remaining 63 contacts (20.5% of 308), for whom insufficient information was available to distinguish between LTBI and TB, were predominantly male (41/63 (65.1%)), born outside Germany (40/54 (74.1%)) and more frequently exposed in community housing (15/63 (23.8%)). Of the 241 contacts with LTBI, 74 initiated preventive treatment (31.6% of the 234 cases with available information about preventive treatment), 66 (28.2%) completed preventive treatment and 197 (81.7%) received a control chest X-ray examination. Six contacts progressed from initially diagnosed LTBI to TB during follow up, amounting to 10 TB diagnoses in total (0.5% of 2,186 contacts). None of these six cases had received preventive treatment, corresponding to a progression rate of 3.6% among the 167 LTBI contacts who had follow-up information available and had not received preventive treatment.

The data contained 124 contacts under 5 years of age. For 94 of these children, a valid TST (25 children) or IGRA (69 children) test was available, while for 23 children no test results were available and seven were lost to follow-up until THT/IGRA testing scheduled at 8 weeks post last exposure to the index patient. In this age group, 72 contacts received a primary prophylaxis (72.7% of the 99 children with available information about this variable) and 21 (16.9%) an initial chest X-ray examination (showing no signs of TB in all cases). *M. tuberculosis* infection was detected in four of the 94 children with a valid test result (4.3%), who were all confirmed to have LTBI and received an initial chest X-ray examination, but had not received primary prophylaxis. Three children with LTBI received preventive treatment and did not progress to TB, while the remaining one did not receive it and progressed to TB.

Testing coverage for *M. tuberculosis* infection was above 90%, irrespective of the contacts’ country of origin and sex, while testing coverage was below 90% in children younger than 5 years (75.8%) or if the exposure setting was community housing (85.0%) or patient/nursing care (83.6%) (Table 3). The highest rates of *M. tuberculosis* infections (> 20%) were observed in contacts born outside Germany (26.2%), or if the exposure settings were community housing (30.1%) or household/family (22.7%).

**Table 3:** Testing coverage, yield and preventive treatment coverage among contact persons of index patients with active pulmonary tuberculosis, selected districts of Upper Bavaria^a^, 2018–2022

Characteristics	CPs tested for *Mycobacterium tuberculosis*			CPs with *M. tuberculosis* infection			CPs with latent tuberculosis who completed preventive treatment		
	n	Total	%	n	Total	%	n	Total	%
All CPs	2,022	2,186	92.5	308	2,022	15.2	66	234	28.2
Country of origin of CP									
Germany	945	1,006	93.9	78	945	8.3	20	60	33.3
Outside Germany	793	863	91.9	208	793	26.2	41	162	25.3
Sex of CP									
Male	1,077	1,181	91.2	187	1,077	17.4	39	136	28.7
Female	944	1,004	94.0	121	947	12.8	27	98	27.6
Age category of CP (years)									
0–4	94	124	75.8	4	94	4.3	3	4	75.0
5–14	107	112	95.5	15	107	14.0	9	13	69.2
15–49	1,247	1,321	94.4	187	1,247	15.0	45	143	31.5
≥ 50	574	629	91.3	102	574	17.8	9	74	12.2
Infectiousness of IP^b^									
Low	714	776	92.0	107	714	15.0	21	79	26.6
Moderate	784	845	92.8	121	784	15.4	31	98	31.6
High	416	456	91.2	67	416	16.1	14	49	28.6
Duration of exposure (hours)									
< 8	262	290	90.3	22	262	8.4	2	14	14.3
8–40	577	631	91.4	64	577	11.1	19	55	34.5
> 40	1,117	1,198	93.2	207	1,117	18.5	45	156	28.8
Exposure setting									
Community housing	209	246	85.0	63	209	30.1	12	45	26.7
Day care / school	172	183	94.0	11	172	6.4	4	10	40.0
Household/family	541	582	93.0	123	541	22.7	34	93	36.6
Patient/nursing care	148	177	83.6	19	148	12.8	2	9	22.2
Social activities	263	288	91.3	35	263	13.3	6	29	20.7
Work	685	706	97.0	57	685	8.3	8	48	16.7

CP: contact person; IP: index patient.

^a^ Bad Tölz-Wolfratshausen, Berchtesgadener Land, Dachau, City of Ingolstadt, Landsberg am Lech, Mühldorf, Pfaffenhofen, Rosenheim, Starnberg and Weilheim-Schongau.

^b^ Infectiousness was categorised as either low (only culture or PCR positive) or moderate/high (smear positive without/with visible caverna/ae on chest X-ray examination).

The proportion of contact persons with LTBI who completed preventive treatment was highest for contacts likely exposed in day care/school (40.0%) or in their household/family (36.6%) as well as in contacts born in Germany (33.3%), and low for contacts exposed at work (16.7%). In particular, preventive treatment coverage differed between children under 15 years (12/17; 70.6%), adolescents and adults aged 15–49 years (31.5%) and adults 50 years and older (12.2%).

Of the 10 contacts with TB, six were born outside Germany, seven were males, five were aged 15–49 years, seven were connected with a moderately or highly infectious index patient and seven had been exposed within their household/family.

### Risk factors for *Mycobacterium tuberculosis* infection in contacts

In a logistic regression analysis based on the 1,570 contacts with available information on all covariates included, the strongest predictors for an *M. tuberculosis* infection in contacts were being born outside Germany (odds ratio (OR) = 2.85; 95% confidence interval (CI): 1.94–4.21) and exposure in community housing (OR = 2.65; 95% CI: 1.65–4.30) or within household/family (OR = 2.62; 95% CI: 1.74–4.00), compared with work as the largest category (Table 4). Significant associations were also observed for age (≥ 50 years), exposure for longer than 40 hours and for a moderate or high infectiousness of the index patient, while sex was not identified as a potential risk factor. The risk estimates were in general similar irrespective of the contacts’ origin, except for community housing, which was associated with infection risk only in contacts born outside Germany.

**Table 4:** Mutually adjusted odds ratios for a *Mycobacterium tuberculosis* infection in contact persons of index patients with open pulmonary tuberculosis disease, selected districts of Upper Bavaria^a^, 2018–2022 (n = 1,570)

Risk factor	All CPs		CPs born in Germany			CPs born outside Germany		
	aOR	95% CI	aOR	95% CI		aOR	95% CI	
CP born outside Germany (reference: in Germany)	2.85	1.94–4.21	NA			NA		
Female CP (reference: male)	1.05	0.77–1.42	0.89	0.54–1.50		1.08	0.73–1.61	
IP born outside Germany (reference: in Germany)	1.20	0.76–1.91	1.25	0.70–2.25		1.55	0.58–4.95	
Age of CP in years (reference: 15–49 years)								
0–4	0.37	0.12–0.93	0.37		0.09–1.15	0.20		0.01–1.07
5–14	0.78	0.41–1.41	0.74		0.28–1.78	0.77		0.31–1.74
≥ 50	1.64	1.14–2.35	1.84		1.03–3.27	1.72		1.06–2.76
Duration of exposure (hours) (reference: < 8 h)								
8–40	1.63	0.82–3.51	0.96		0.34–3.00	2.26		0.86–7.16
> 40	2.34	1.23–4.87	2.77		1.16–7.93	2.31		0.92–7.09
Exposure setting (reference: work)								
Community housing	2.65	1.65–4.30	0.37		0.00–3.14	2.87		1.62–5.24
Day care / school	0.69	0.32–1.40	1.16		0.34–3.40	0.47		0.16–1.21
Household/family	2.62	1.74–4.00	2.46		1.26–4.86	2.79		1.59–5.04
Patient/nursing care	0.85	0.26–2.22	0.75		0.19–2.31	0.85		0.04–5.70
Social activities	1.39	0.78–2.44	1.02		0.36–2.59	1.85		0.86–3.92
Infectiousness of IP (reference: low)								
Moderate	1.78	1.27–2.51	1.45		0.81–2.64	1.94		1.26–3.00
High	1.61	1.09–2.37	1.57		0.75–3.22	1.53		0.96–2.43

aOR: adjusted odds ratio; CI: confidence interval; CP: contact person; IP: index patient; NA: not applicable.

^a^Bad Tölz-Wolfratshausen, Berchtesgadener Land, Dachau, City of Ingolstadt, Landsberg am Lech, Mühldorf, Pfaffenhofen, Rosenheim, Starnberg and Weilheim-Schongau.

**Figure 2:**
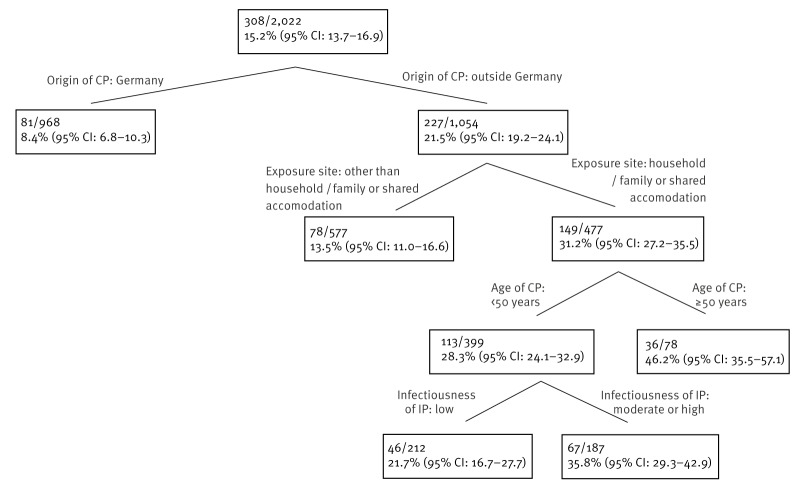
Classification tree for prevalence of *Mycobacterium tuberculosis* infection in contact persons of index patients with active pulmonary tuberculosis, selected districts in Upper Bavaria^a^, 2018–2022 (n = 2,022)

CI: Confidence interval; CP: contact person; IP: index patient.

^a^ Bad Tölz-Wolfratshausen, Berchtesgadener Land, Dachau, City of Ingolstadt, Landsberg am Lech, Mühldorf, Pfaffenhofen, Rosenheim, Starnberg and Weilheim-Schongau.

For the classification tree analysis, all 2,022 contacts who had information about *M. tuberculosis* infection available were eligible for the analysis. Starting with an initial prevalence for *M. tuberculosis* infection of 15.2%, the classification tree stratified infection risk by a factor of 5.5, as the prevalence in contacts born in Germany was 8.4% (95% CI: 6.8–10.3) compared with a prevalence of 46.2% (95% CI: 35.5–57.1) in contacts born outside Germany, exposed in their household/family or in a shared accommodation, and aged 50 years or older (Figure 2).

Data are shown with prevalences and corresponding 95% binomial confidence intervals (in brackets). Missing values of predictor variables were imputed using surrogate variables.

## Discussion

Our evaluation of TB contact investigation in 10 districts in Upper Bavaria, Germany, revealed that a median of 7.5 contacts per index patient were followed up, of which more than 90% were tested for *M. tuberculosis* infection. Of these, 15% were infected and 10 contacts (0.5%) developed TB. Fewer than 30% of those identified with LTBI received preventive treatment.

The median number of contacts investigated aligns with prior German data [10], but was higher than in other low-incidence European countries [20,21]. This discrepancy may be underestimated, as contacts living outside the index patients’ residential district were not included in our data. This observation may reflect a more inclusive contact tracing strategy in Germany. While this may reduce the risk of missing secondary cases, it is also more resource-intensive and may reduce the yield. Reinforcing the close contact selection criteria, e.g. through medical training programs, may help to ensure proportionality of measures and resources. For a broader yet efficient contact tracing approach, the ‘stone in the pond’ principle may be applied, which comprises comparing the infection yield among close contacts with expected age and origin-specific background prevalence and expanding investigations to more casual contacts only if the yield exceeds background levels [6].

High testing coverage among contacts indicates overall good guideline compliance. However, coverage was somewhat lower in community housing or patient/nursing care settings, indicating the need to identify and address setting-specific barriers. Also, there was insufficient information to distinguish between LTBI and TB due to missing chest X-ray results for 20.5% of cases, the majority of whom were born outside Germany. We speculate that patient relocation or low risk awareness may have been among the reasons for this. For children younger than 5 years, we found considerable deviation from German guidelines recommending prompt screening as well as primary prophylaxis. Valid TST/IGRA testing results were available for only 75.8%, and only 16.9% received an immediate chest X-ray. Similarly low TST/IGRA testing and chest X-ray coverage for children below the age of 5 years was found in Switzerland [22]. The low chest X-ray coverage suggests limited awareness among healthcare providers of the risk of disease progression within the pre-allergic phase in children younger than 5 years and hence the indication for immediate chest X-ray examination independent of TST/IGRA results [23]. Primary prophylaxis coverage was 72.7% among children younger than 5 years, lower than previously reported for contact investigations in Bavarian day care settings [24] and similar to figures from Switzerland [22]. This may reflect individual recruitment delay beyond the pre-allergic phase, medical contraindications, or parents’ reluctance towards prolonged administration of antibiotics. Four children were diagnosed with LTBI, all of them in absence of primary prophylaxis. One child with LTBI who had also received no preventive treatment progressed to active TB during follow-up. Taken together, our findings underscore the need to strengthen the implementation of contact tracing guidelines for this highly vulnerable age group.

The observed yield of 15.2% of *M. tuberculosis* infection among contacts aligns with previous findings from Germany and some other low-incidence settings [8-10,21,25,26], but was notably lower than the pooled yield of 28.6% reported for high-income, low-incidence settings [7]. The difference may result from variations in the background prevalence and/or incidence of *M. tuberculosis* infection or from differing policies on contact eligibility, enrolment, diagnostic testing or duration of follow-up. Notably, a historically more widespread use of TST, which tends to overestimate infection status, may contribute to this discrepancy. Our yield of active TB disease was 0.5% and lower than previously reported for high-income, low-incidence settings (1.9%) [7], London (1.8%) [20], the Netherlands (0.7%) [21] and the metropolitan area of Torino (0.7%) [27]. We observed a significantly higher yield of *M. tuberculosis* infection among contacts born outside Germany (26.2%) than German-born contacts (8.3%), consistent with previous studies in Germany and other low-incidence countries [9,27-29]. A substantial proportion of LTBI among contacts born outside Germany may stem from prior exposure in their country of origin rather than recent transmission from the index case [30]. Similarly, the higher yield observed among older contacts, as reported in previous studies [9,10,31], likely reflects long-standing infections with a lower risk of progression. In contrast, LTBI in children is more indicative of recent transmission and should therefore be prioritised for identification and treatment. Further independent risk factors for *M. tuberculosis* infection in close contacts included contagiousness of the index patient, cohabitation/ household/ first-circle contact and prolonged exposure consistent with previous German studies [9,10] and studies in other low TB incidence countries [26,27,31,32].

Our classification tree analysis indicates that the consideration of only two factors – contact origin and exposure setting – may double the detection rate of *M. tuberculosis* infections (31.2% vs 15.2%). However, this gain in efficiency resulted in the detection of only around half of the infections (149/308). With almost 50%, implying a threefold increase in efficiency, the highest prevalence of *M. tuberculosis* infection occurred among contacts born outside Germany exposed in household/family or shared accommodations and aged 50 years or older. However, this subgroup contained only 10% (36/308) of all infected contacts. These results provide a potential approach for increasing efficiency in contact investigations in similar settings for times when the contact tracing capacities are limited (e.g. under pandemic conditions) and an estimation of how many infected cases might be missed with this approach.

Preventive treatment coverage among LTBI contacts in our study was notably low, indicating potential gaps in treatment uptake or provider engagement. Coverage differed by age, being higher among children and particularly low among adults 50 years or older. The latter reflects recommendations before 2023 restricting the use of isoniazid-based preventive treatment in this age group due to potential side effects [13]. Reassuring safety data for rifampicin-based regimens have since prompted authorities to extend recommendations for chemoprevention to older adults [23]. Future studies may assess the effect of the revised guideline. Coverage was also lower among contacts born outside Germany. This may reflect physicians’ uncertainty regarding the risk-benefit ratio of preventive treatment in individuals with long-standing LTBI, compared with those with recently acquired infections. Contacts from high-incidence countries may indeed have long standing TB infection acquired in their country of origin [29]. However, coprevalent and incident LTBI are indistinguishable in practice and may coexist. Therefore, German guidelines recommend preventive treatment for all recently exposed individuals diagnosed with LTBI, even if previous LTBI is plausible [33]. The use of a risk assessment score, such as the one described by Saunders et al. [34], could help select contacts with LTBI who would benefit from preventive treatment and minimise the number needed to treat [35]. Coverage was particularly low among contacts exposed at work, indicating the need for stronger collaboration with occupational medical services. Interestingly, coverage was higher among contacts of highly infectious index patients, suggesting that the proximity with a seriously ill index case and the perceived risk of disease progression may positively influence treatment uptake. Overall, identifying and addressing the underlying reasons for the low preventive treatment coverage is crucial to preventing the progression from LTBI to active TB and achieving TB elimination goals.

To our knowledge, the present study provides the first comprehensive evaluation of TB contact investigation in Germany, assessing not only the yield of *M. tuberculosis* infection but also key indicators such as testing and preventive treatment coverage. Our analysis is based on a large, recent dataset from 2018 to 2022, covering TB cases and their contacts in a Bavarian region with ca 1.5 million inhabitants. Cases in this area were largely comparable to cases in the rest of Bavaria, even though they had slightly higher proportions of persons born outside Germany and of MDR/pre-XDR drug resistance. Additionally, we evaluated a broad range of index patient and contact characteristics, which were often not all included in previous studies. Finally, the classification tree analysis offers an automated, unbiased and easily interpretable statistical approach to split a given dataset into low- and high-risk groups, taking potential interactions between the predictor variables into account.

However, one limitation of our study is that we did not have detailed data on contacts living outside the index patient’s district of residence. The available information indicated that we underestimated the number of contacts per index patient by a median of four contacts. As the missed contacts predominantly were non-household contacts, we possibly overestimated the yield of *M. tuberculosis* infection. Moreover, not all cases notified to the LGL were also included in the study. Apart from notification errors, this may probably reflect that some index cases did not report any close contacts, as index cases were only included in this study if they had at least one associated close contact who was traced by the same local public health department.

## Conclusion

Tuberculosis contact investigation in Upper Bavaria is marked by a high screening coverage of contacts and a *M. tuberculosis* infection yield and risk factors comparable to other low-incidence settings. However, there were gaps in the implementation of guidelines for preventive treatment and the screening of children under 5 years of age. Future research should explore the underlying causes of these gaps. Our findings may inform targeted interventions such as educational campaigns for healthcare providers. Overall, our indicator estimates contribute to the monitoring and evaluation of TB contact investigation in Germany. Ideally, aggregated information on TB contacts should be included into the national surveillance system, allowing the continuous and comprehensive monitoring and evaluation.

## Data Availability

Due to data protection issues, the data cannot be made publicly available. The analysis code deposited at the time of publication, as well as official population numbers for Bavaria as of 2022 (in German, as published by the Bavarian State Statistical Office), are available at https://osf.io/4ztxy/.

## Supplementary Data

251000 Supplementary Material
