# Supplementary material for: Tuberculosis contact investigation: an evaluation of yield and guideline adherence, Upper Bavaria, Germany, 2018 to 2022
Source: Euro Surveill. 2025 Oct 2;30(39):2500096. doi: 10.2807/1560-7917.ES.2025.30.39.2500096 (PMC12495383; doi:10.2807/1560-7917.ES.2025.30.39.2500096)
Supplement: Supplementary Material [file 25-00096_SCHMIDT-OTT_Supplement.pdf]

Supplementary material

This supplementary material is hosted by Eurosurveillance as supporting information alongside the article "Tuberculosis contact investigation: an evaluation of yield and guideline adherence, Upper Bavaria, Germany, 2018 to 2022", on behalf of the authors, who remain responsible for the accuracy and appropriateness of the content. The same standards for ethics, copyright, attributions and permissions as for the article apply. Supplements are not edited by Eurosurveillance and the journal is not responsible for the maintenance of any links or email addresses provided therein.

Supplementary Table S1 was incorrect at the time of publication, as some of the footnotes were not assigned appropriately. The table was corrected and replaced on 21 May 2026.

Supplement S1. Criteria for being classified as a close contact in centrifugal tuberculosis contact investigations according to German guidelines (1)

Individuals were classified as close contacts based on the following criteria:

- Living in the same household, room (e.g., in a care facility, hospital, or prison), or other enclosed spaces as the index case
- Intimate or other close physical contact in enclosed spaces, even if singular, e.g.:
    - close physical contact (e.g., dancing, contact sports)
    - singing together in a choir
    - intimate contact
    - caregiving activities
    - medical procedures, including oral, dental or otolaryngological examinations, sputum induction, open-system suctioning of the nasopharynx, bronchoscopy, resuscitation procedures and autopsy procedures
- Cumulative exposure to the index case in enclosed spaces:
    - ≥ 8 hours for a smear-positive index patient
    - ≥ 40 hours for a smear-negative but culture- or PCR-positive index patient

Priority groups for contact investigation include children, adolescents, and immunosuppressed individuals, as they have a higher risk of developing active TB following infection. Individuals who do not meet the above criteria should only be included in the investigation after careful assessment of their individual disease risk.

Supplementary Table S1. Comparison between index patients with active pulmonary tuberculosis in contact investigations of selected districts in Upper Bavaria* and pulmonary tuberculosis cases** notified to the Bavarian Health and Food Safety Authority between 2018 and 2022.

	**Study Index Cases**		**Notified Patients in Bavaria**		**p-value**[1]
	n	%	n	%	
Total	171	100.0	2097	100.0	
Country of birth					0.047
Germany	39	23.1	619	29.8	
Outside Germany	130	76.9	1461	70.2	
Unknown	2	NC	17	NC	
Sex					0.596
Male	115	67.3	1450	69.4	
Female	56	32.7	640	30.6	
Unknown	0	NC	7	NC	
Age					0.254
0-4 years	1	0.6	17	0.8	
5-14 years	1	0.6	18	0.9	
15-49 years	91	53.5	1254	59.8	
≥50 years	77	45.3	808	38.5	
Unknown	1	NC	0	NC	
Sputum smear microscopy of respiratory material[2]					0.891
Positive	100	59.2	1239	60.0	
Negative	69	40.8	825	40.0	
Unknown	2	NC	33	NC	
PCR of respiratory material[2]					0.661
Positive	140	87.0	1665	87.9	
Negative	21	13.0	229	12.1	
Unknown	10	NC	203	NC	
Culture of respiratory material[2]					0.432
Positive	161	96.4	1951	95.0	
Negative	6	3.6	103	5.0	
Unknown	4	NC	43	NC	
Drug resistance					0.007
Sensitive	150	93.2	1734	96.5	
MDR	7	4.3	52	2.9	
pre-XDR	4	2.5	11	0.6	
Other/ unknown	10	NC	300	NC	

MDR: multidrug resistant, NC: not calculated, pre-XDR: pre-extensively drug-resistant.

*Bad Tölz-Wolfratshausen, Berchtesgadener Land, Dachau, City of Ingolstadt, Landsberg am Lech, Mühldorf, Pfaffenhofen, Rosenheim, Starnberg and Weilheim-Schongau

**confirmed pulmonary tuberculosis patients with positive smear microscopy, culture and/or PCR of respiratory material (sputum, bronchoalveolar lavage specimens, bronchial secretion or gastric juice)

[1] Pearson's Chi-squared test; Fisher's exact test; comparison of study index cases with cases notified in Bavaria excluding those notified in districts participating in the study

[2] sputum, bronchoalveolar lavage specimens, bronchial secretion or gastric juice

Percentages denote column percent of non-missing values.

Supplementary Table S2. Exposure sites of contact persons transferred to other districts of index patients with active pulmonary tuberculosis in contact investigations in 2018-2022 of selected districts in Upper Bavaria*.

	**N**	**%**	**Median per IP (IQR)**
Total	759	100.0	4 (2, 8)
Exposure site			
Community Housing	28	3.7	0 (0, 0)
Day care / School	189	25.0	0 (0, 0)
Family	96	12.7	0 (0, 2)
Patient / Nursing care	70	9.3	0 (0, 1)
Social activities	41	5.4	0 (0, 0)
Work	332	43.9	0 (0, 3)
Unknown	3	NC	0 (0, 0)

IP: index patient, IQR: interquartile range, NC: not calculated

* Bad Tölz-Wolfratshausen, Dachau, Landsberg am Lech, Mühldorf, Pfaffenhofen, Rosenheim, Starnberg and Weilheim-Schongau

Percentages denote column percent of non-missing values.

References

1. Diel R, Loytved G, Nienhaus A, Castell S, Detjen A, Geerdes-Fenge H, et al. [New recommendations for contact tracing in tuberculosis. German Central Committee against Tuberculosis]. Pneumologie. 2011;65(6):359-78.
